# Eleven quick tips for organizing a data cleaning challenge

**DOI:** 10.1371/journal.pcbi.1013791

**Published:** 2025-12-16

**Authors:** Ireen J. Kal, Lidwien P. Smabers, Joanna von Berg, Giulia Perticari, Mette van de Meent, Denise Fokkelman, Joep Sprangers, Loïc Lannelongue, Florijn Dekkers

**Affiliations:** 1 Princess Máxima Center for Pediatric Oncology, Utrecht, The Netherlands; 2 Department of Medical Oncology, University Medical Center Utrecht, Utrecht University, Utrecht, The Netherlands; 3 Department of Obstetrics, Wilhelmina Children’s Hospital, Utrecht, The Netherlands; 4 Department of Strategy and Policy, Program Sustainable Healthcare, University Medical Center Utrecht, Utrecht, The Netherlands; 5 Center for Molecular Medicine, University Medical Center Utrecht, Utrecht University, Utrecht, The Netherlands; 6 Cambridge Sustainable Computing Lab, Department of Public Health and Primary Care, University of Cambridge, Cambridge, United Kingdom; 7 British Heart Foundation Cardiovascular Epidemiology Unit, Department of Public Health and Primary Care, University of Cambridge, Cambridge, United Kingdom; 8 Victor Phillip Dahdaleh Heart and Lung Research Institute, University of Cambridge, Cambridge, United Kingdom; 9 Health Data Research UK Cambridge, Wellcome Genome Campus and University of Cambridge, Cambridge, United Kingdom; 10 Education Center, Faculty of Medicine, University Medical Center Utrecht, Utrecht University, Utrecht, The Netherlands; Montreal, CANADA

## Introduction

The exponential growth of data continues to revolutionize research methodologies. These technological advancements enhance data accessibility, improve collaboration, and accelerate scientific discoveries [1,2]. Particularly in healthcare, data—for instance from whole genome sequencing—is accumulating rapidly [3]. Novel computational resources and tools are allowing researchers to analyze large volumes of medical data. This leads to more precise diagnostics, personalized treatments, and better patient outcomes [1,2].

At the same time, concerns about the environmental impact of data storage and computing are growing [4,5]. Data centers, which are essential for processing and storing data, consume significant energy and water, emit greenhouse gases, and have broad ecological impacts [6]. The expansion of data centers often contributes to land use changes, biodiversity loss, and social challenges [6]. Addressing these issues requires practical tools and protocols across academic, healthcare, and organizational settings.

Another profound issue is the limited reproducibility of research leading to unnecessary follow-up studies and an increased carbon footprint [7]. Such lack of reproducibility could be partly limited by following the Findable, Accessible, Interoperable, and Reusable (FAIR) data principles [8] and proper data management to avoid wasted research efforts.

**Fig 1:**
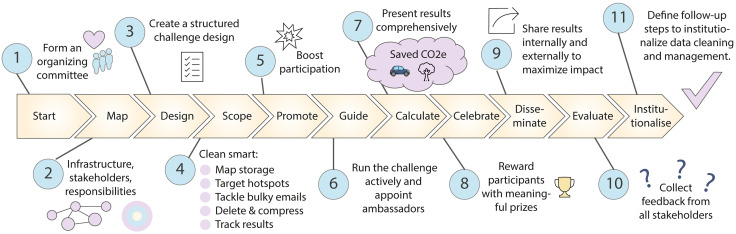
Graphical abstract of 11 quick tips to get started with organizing a data cleaning challenge within an organization, including hands-on tips for individual researchers aiming to clean up data effectively (Tip 4).

Inspired by the success of freezer challenges to reduce the environmental footprint of frozen sample storage in labs [9,10], we developed the data cleaning challenge. This initiative encourages staff to delete unnecessary data and improve data management, thereby lowering the environmental impact of data storage. We present a roadmap with 11 quick tips (Fig 1) for organizing a data cleaning challenge within any organization.

**Fig 2:**
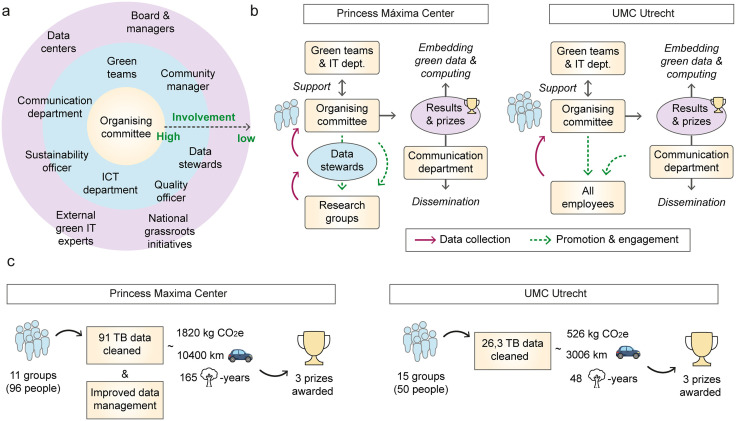
Set up and results of two data cleaning challenges held in the Netherlands. **(a)** Example of a stakeholder map generated to guide the organization. **(b, c)** Schematic overview of the set-up (b) and result highlights (c) of data cleaning challenges of the Princess Máxima Center and UMC Utrecht.

This concept has been developed based on insights from two challenges we organized in Utrecht, The Netherlands, each with a different approach (Fig 2 and Table 1). These efforts led to substantial reduction in data stored (together more than 100 terabyte (TB) cleaned), enhanced data management practices, and further institutionalization of sustainable data handling (Fig 2 and Table 1 and S1 and S2 Text files).

**Table 1:** Overview of information on data cleaning challenges held at the Princess Máxima Center and UMC Utrecht.

**Institute**	Princess Máxima Center Pediatric cancer hospital with embedded research department.	UMC Utrecht Large academic hospital combining healthcare, research and education.
**Institute characteristics**	Total number of staff: > 1,500 Number of research groups: 36 Number of staff at research department: 495 Number of participating groups: 11 Number of participating individuals: 96	Total number of staff: >12,000 Departments: 11 Number of participating groups: 15 Number of participating individuals: 50
**Challenge approach**	Research groups, actively engaging data stewards.	All employees, no targeted data hotspots.
**Organizing committee**	Three green team (Tip 2) research members (with bioinformatics expertise). Time investment: 12 hours per person in total	Three green team (Tip 2) research members, community manager sustainability, and sustainability expert. Time investment: 6 hours per person in total
**Stakeholders**	ICT department, sustainability officer, data stewards, communications department, green team research, and Green Labs NL Foundation.	ICT department, data board for changing data-policy, data storage team, division data managers meeting, team Office 365, green team research, communications department.
**Challenge design**		
*Audience*	Clinical & pre-clinical research groups.	All staff.
*Duration of the challenge*	1 month.	2 months.
*Scope*	Compute and storage clusters, personal folders, emails.	Personal folders, emails.
*Process*	Team signup → Instructions → Data cleaning → Results collected (S3 and S4 Text files).	Instructions → Data cleaning → Results collected (S3 and S4 Text files).
*Communication*	Emails, intranet posts, meetings, posters, social events.	Intranet, posters, newsletters, ICT sub-departments, Green Teams.
*Timeline*	Promoting announcements 2 weeks before, promoting posters 1 week before, reminders of participation every 2–3 weeks during the challenge.	Promoting announcements 2 weeks before, promoting posters 1 week before, reminders of participation every 2–3 weeks during the challenge.
**Results**		
*Data collection*	Google Forms	Google Forms
*Prizes*	1) Highest absolute data storage reduction, 2) Highest relative data storage reduction, 3) Best data management.	1) Largest team, 2) Highest absolute data storage reduction, 3) Highest average data storage reduction per member.
*Communication*	Prize ceremony, intranet articles, national Green Labs NL Foundation meeting.	Prize ceremony, email updates, newsletter, LinkedIn, national PhD event.
*Evaluation*	Feedback from data stewards & green team research.	Increased awareness of data storage impact.
**Follow up**	Encouraged research groups to host their own data clean-ups. Organized two data steward meetings on data cleaning strategies. Established a green IT team within the green team research.	Developed recommendations for removing old and untraceable data as follow-up project and ICT policy improvements.

Our roadmap offers practical guidance and flexible strategies for organizations looking to reduce the environmental footprint of their data practices. It also provides hands-on tips for individual researchers aiming to clean up data effectively (Tip 4). Through this initiative, we aim to empower both individuals and institutions to proactively address the environmental challenges of digitalization in science and healthcare. While the methods described in this article were inspired by data cleaning challenges in research-performing hospitals, the principles can be adopted in other fields with domain-specific adaptations.

## Tip 1: Form a passionate, cross-functional organizing committee

The first step is to assemble an enthusiastic organizing committee to steer the initiative. Both challenges in the Netherlands were a bottom-up initiative from the green team of the research department, sparked by organizing a green IT-themed internal meeting. Preferably, the committee would consist of people with diverse roles and expertise. The committee would benefit from members with proficiency in big data analytics, advanced computing, or who are working in the Information and Communications Technology (ICT) department. In addition, it is recommended to include individuals who understand the governance of the organization and are passionate about sustainability or data organization. The committee is responsible for the overall management of the challenge, including challenge design, communication strategy, result processing, evaluation, and celebration of successes. To generate enthusiasm and engagement, a seminar on green data management and computing could be hosted. Consider inviting an external speaker who is expert on this topic.

## Tip 2: Map the institution’s infrastructure, identify stakeholders, and assign clear responsibilities

To maximize the impact of the challenge, it is key to understand the institute’s governance and data infrastructure and pinpoint critical stakeholders who can offer advice or support (Fig 2a). Stakeholders with high involvement include:

1. The **ICT department** may give insight into the data storage systems and provide facts and figures on data storage and energy consumption. Furthermore, they could help to identify data storage hotspots (groups that store large volumes of data) and help with general policies for data management and cleaning. Typically, data management policies include details on how project data will be handled across the full research data life cycle (collecting, storing, using, sharing, archiving, and re-using of data). When closing a project, it is important that all raw and processed data needed to reproduce the results are kept, whereas unnecessary (intermediate) files should be cleaned up. Data management policies often also have information on how General Data Protection Regulation principles, including storage requirements and limitations, will be applied in the project. The ICT department could provide access to online tools, such as DMP online [11], a platform for sharing data management policies.
2. **Green teams**, typically a group of employees that work on initiatives to reduce the environmental impact of the organization, are often most passionate about sustainability and reducing energy consumption. They could help organize the challenge and encourage people to participate.
3. **Data stewards** or **quality officers** usually coordinate data management among bigger groups and could function as key players between participants and the organizing committee.
4. Professionals with **dedicated roles in sustainability**, such as community managers or sustainability officers could use their network to promote the challenge. Additionally, they may lobby for further institutional changes after the challenge.
5. **The communication department** could support outreach and communicate achievements of the challenge internally and externally.

In addition, external stakeholders with lower expected involvement can still play a valuable role (Fig 2a). Informing **internal managers or board members** ensures institutional awareness, while engaging with external green science **grassroots initiatives** could support national dissemination. Besides, connecting with the **data center** responsible for the institute’s stored data would help to retrieve environmental impact metrics on data storage. Other valuable contacts include *Data Champions*, who are employees appointed by some institutions to advise researchers on best practices for handling research data [12]. Importantly, tips and tricks can be gathered from organizations with prior experience in data cleaning challenges.

## Tip 3: Create a structured, well-scoped challenge design

A well-structured design is essential for an impactful challenge. The following points could be considered:

- Target audience: determine the level of participant involvement, such as the individual, group, or department level. The Princess Máxima Center specifically targeted research groups, while the University Medical Center (UMC) Utrecht invited all employees to participate individually (Table 1).
- Duration: estimate how much time the participants would need for data cleaning and documentation of their cleaning activities.
- Scope of cleaning: make an inventory of storage entities used by the target audience in the organization and define which systems should be cleaned, such as shared storage and/or personal storage.
- Result collection: provide a format in which participants should collect and submit results, such as Excel templates or digital forms. The forms should include clear instructions on how to clean data for different storage entities to ensure uniform input. Example forms are accessible in S3 and S4 Text files.
- Evaluation criteria: determine the factors for evaluating cleaning efforts and awarding of points. For example, participants at the Princess Máxima Center were asked to record stored data sizes before and after cleaning across five different storage entities, enabling the calculation of absolute and relative cleaned data amounts (S3 and S4 Text files and Table 1). Additionally, data management strategies were evaluated, including the existence of data management plans for research projects. This also involved data exit strategies, typically an instructive document that ensures departing group members clean up their data. In contrast, the UMC Utrecht challenge focused solely on cleaning data from three storage entities (S3 and S4 Text files and Table 1).
- Winner selection: decide the number of winners and the categories in which prizes will be awarded.

## Tip 4: Clean smart: Focus on high-impact storage for maximum efficiency

***For the organizing committee of a challenge:***

- Start with thorough inventory of storage entities used by the target audience. During the challenges, this inventory was done by the organizers. This ensures that no relevant locations are overlooked and enables the development of data collection forms that are appropriately tailored. Examples of such entities include personal folders on personal or shared drives, shared folders, and email inboxes.
- Be cautious with instructions on cleaning emails. Most are lightweight and deleting them may consume more energy than keeping them. Therefore, instruct participants to sort their emails by size and encourage them to delete large emails to quickly free up storage space. A good alternative is sorting emails by sender, such as newsletters, allowing entire threads from the same source to be deleted efficiently.
- To ensure the most effective data cleaning, storage ‘hotspots’ within the organization should be identified with the help of the ICT team. This way, research groups working with large data files, or specific departments or facilities, can be targeted. The terminal command “du -h -d 1” can be used to check disk usage. By developing a strategic plan to actively engage employees in these areas, the impact of the cleanup effort can be increased.
- In addition to deleting data, encourage the compression of files and folders (for example using zip or gzip) as a means of optimizing storage space. It should be noted that compressing data does increase CPU load, so the optimal balance depends on frequency of usage and system energy profile. Compression is generally most effective for large datasets, reducing file sizes by 10–100× and input/output energy by up to 25% in high-performance computing systems [13].

***For individual researchers:***

- Map your storage landscape to identify personal data hotspots (e.g., folders with large raw datasets and old project backups). Start with the biggest files and folders.
- Employ available tools or consult a data steward to detect duplicates and redundant versions of files (e.g., PowerPoint backups).
- When tackling email, apply the same principles: prioritize large attachments (by sorting on size) or mass threads, rather than bulk deletion.
- Track the size of storage entities before and after cleaning to demonstrate your impact, for both personal reflection and peer comparison. Use environmental impact estimates (Tip 7) to quantify results and share your outcomes to inspire others.

***Tiered data storage (‘hot’ versus ‘cold’ storage):***

- Store frequently accessed (‘hot’) data on high-speed media, such as solid-state or hard disk drives, to ensure rapid retrieval and response times. Place infrequently accessed (‘cold’) data on more sustainable storage options, such as removable drives or compressed archives [14].

## Tip 5: Boost participation by highlighting data impact and announcing prizes early

To effectively promote the challenge, a communication plan tailored to the institution is essential. The plan should detail multiple means of communication, timelines, actions, and responsibilities. It should include approaches to engage the target audience to participate, award the prizes, and disseminate challenge results (Tips 8 and 9). Utilizing existing communication channels, meetings, and events can enhance outreach. In the Princess Máxima Center and the UMC Utrecht, the challenges were promoted through intranet, newsletters, meetings, events, flyers, and reminder emails (Table 1). To raise awareness and encourage participation, numbers can be provided on the environmental, financial or social impact of data storage, such as those published previously [6,15,16]. Lastly, it is recommended to announce prize categories in advance, to boost engagement by clarifying how winners will be chosen.

## Tip 6: Guide participants: Run the challenge actively and appoint ambassadors

When running the challenge, consider appointing challenge ambassadors to drive engagement and support participants. In addition to the organizing committee, challenge ambassadors could include data stewards, community managers, or green team members. The duration of the two Dutch data cleaning challenges was one or two months (Table 1), allowing flexibility to allocate time for data cleaning. The optimal timeline may differ, depending on the type of institute and target audience. During this period, both organizing committees sent reminder emails to encourage participation, answered participant questions, and confirmed when participants submitted their results (Table 1). In the Princess Máxima Center, the data stewards guided their team members and collected results. In the UMC Utrecht guidance was provided by the organizing committee.

## Tip 7: Use environmental impact metrics to present results comprehensively

After the challenge, the organizers can calculate the volume of cleaned data per storage entity and determine the winners (Tip 8). To increase reproducibility and accountability, organizers can either give instructions on how exactly to remove data and record storage, or request participants to share their methodologies. Examples of instructions can be found in S3 and S4 Text files. To make results comprehensive for a broad audience, organizers could express cleaned data as environmental impact, such as saved greenhouse gas emissions expressed as the equivalent amount of carbon dioxide (CO₂) saved (CO₂-equivalent or CO₂e). However, the environmental impact of data storage for specific institutions is often unclear, as it depends on factors such as the data center’s energy source and is complex for large institutions with data stored across multiple locations. The carbon footprint of storing data is assumed to be in the order of 10 kg CO₂e per TB per year [4,16]. Assuming that all data is backed up at least once, we used a value of 20 kg CO₂e per TB of data for calculating the carbon footprints in the challenges (Fig 2). Additionally, to express environmental impact, CO₂e was converted to kilometers driven by a European car and CO₂ sequestered by trees per year (tree-years), using metrics developed previously [17].

The following examples show how results can be communicated in a way that is meaningful to a broad audience (Fig 2c). At the Princess Máxima Center, 96 participants from 11 research groups removed a total of 91 TB of data. This can be expressed as 1,820 kg CO₂e per year, 10,400 km driven by a European car. Alternatively, it represents the annual sequestration of CO₂ by 165 trees, and is in the range of 15,000 euros per year. Ensure that besides quantitative data (e.g., storage cleaned), qualitative results (e.g., data management practices) are also measured to gain a comprehensive view of the impact. In the Princess Máxima Center, 82% of groups implemented data exit strategies for departing staff, 73% of the groups maintained data records and management plans (Tip 2) for ongoing projects, and 45% of groups compressed data during cleaning (Fig 2c; S1 and S2 Text files). At UMC Utrecht, 50 participants from 15 groups removed 26.3 TB of data, corresponding to 526 kg CO₂ per year, 3,006 km driven by a European car, or the sequestration of CO₂ by 48 trees annually (Fig 2c; S1 and S2 Text files). Additionally, efforts are made to implement a Standard Operating Procedure for data storage, including guidelines on how to minimize the environmental impact of data storage. Lastly, a dialogue between the UMC Utrecht and its data center was initiated to obtain metrics on environmental impact measures for stored data.

These results demonstrate that the challenges led to reducing data storage requirements which can lower CO₂ emissions and costs over time. By eliminating redundant, outdated, or irrelevant data, and improving data management, organizations can enhance research reproducibility and optimize their existing storage infrastructure, delaying the need for additional storage expansion. Thereby, data cleaning also limits production of new hardware, leading to less CO₂ emission related to hardware life cycle, known as embodied carbon. However, it is important to critically assess the long-term impact. While data cleaning can slow down the rate at which storage capacity needs to be increased, it does not necessarily lead to an immediate reduction in energy consumption. Data centers typically operate at a fixed energy demand. This implies that, unless storage infrastructure is actively downsized or decommissioned, the overall energy savings may be limited in the short term.

## Tip 8: Celebrate successes and reward participants with meaningful prizes

Positive feedback and celebration of achievements raise further awareness and can motivate individuals and communities to engage in collective actions for sustainable development. The Princess Máxima Center and UMC Utrecht awarded prizes in 3 categories (Table 1). Eco-friendly prizes align with the environmental focus of the challenge. Possible prize categories include biggest absolute difference in data storage, biggest relative difference in data storage, highest average data storage reduction per member and/or best data management practices. The prizes can be distributed at an award ceremony at a scheduled event that has a broad audience to maximize exposure. Photos of the winners with their award can be taken for communication purposes.

## Tip 9: Disseminate results internally and externally to maximize impact

Follow the communication plan (Tip 5) for disseminating results within the institute and to the wider community to raise awareness, receive feedback, and inspire others to organize a similar challenge. The communications department can support the effective dissemination of results. Both Dutch organizing committees disseminated results with support from the communication department. This was done through various channels. Examples include an email to all participants, an article on intranet or in the internal newsletter, and social media (Table 1). In addition, both teams were invited to present their results at external events, including at a national green science community meeting by Green Labs NL Foundation [18]. Use storytelling to make results more engaging. Highlight personal experiences from participants and share photos of the organizing team or data cleaning winners.

## Tip 10: Evaluate the challenge by collecting feedback from all stakeholders

It is important to analyze the results and evaluate the process in collaboration with various stakeholders, such as challenge participants. Both successful aspects, obstacles and potential strategies to tackle them could be discussed.

At the Princess Máxima Center, a high participation rate (31% of research groups, with 55% of members actively cleaning data) and substantial data reduction (S1 Text) were likely driven by several factors. The research department’s structured data stewardship—where each team has a dedicated steward—and regular data steward meetings facilitated the coordination. Additionally, the visibility of the green team and prior sustainability initiatives, like a freezer challenge, heightened awareness. ICT’s support in identifying high-volume storage users also contributed to efficiency. Small department size further streamlined communication between the green team, data stewards, and communication teams.

At UMC Utrecht, outreach efforts faced some challenges due to the institution’s larger size, entailing that identification of the right stakeholders and the establishment of personal engagement strategies proved more difficult. The fact that only a few data ambassadors (e.g., data stewards) were dedicated to managing data collection likely contributed to lower data-cleaning volumes. However, engaging ICT staff in the process helped raise awareness about sustainability, eventually influencing policy changes within the department.

## Tip 11: Define follow-up steps to institutionalize data cleaning and management

The organization of a data cleaning challenge could kickstart regular data cleaning efforts and improvement of data management. It would be useful to appoint stakeholders as ambassadors for future data cleaning challenges. Early involvement of change-makers, such as sustainability officers or data stewards, helps embedding data cleaning challenges in long-term sustainability efforts. As follow-up steps, the UMC Utrecht aims to organize a yearly data cleaning challenge. Furthermore, the ICT department was provided with recommendations on the reduction of data stored by default. For the challenge at the Princess Máxima Center, research groups were encouraged to organize yearly data cleaning challenges themselves. Also, two data steward meetings focused on data cleaning strategies (Table 1). Finally, the Dutch Federation of University Medical Centers is currently performing a project to increase sustainability within laboratories, which includes a data cleaning challenge in all University Medical Centers.

## Conclusion and outlook

Our data cleaning challenges have demonstrated that targeted initiatives can significantly reduce data storage footprints in the long term, while raising awareness about the environmental impact of digital storage. This concept contributes to the growing toolkit for greening computational science. Examples of other tools and resources are GreenDisc [19], a certification tool for sustainable data management launched in 2024, impact calculators like Green Algorithms [20], and coding packages such as CodeCarbon [21] and CarbonTracker, which monitor electricity usage over time. Additionally, the *Environmentally Sustainable Computational Science* community platform [22] connects like-minded researchers and facilitates knowledge sharing in this field. The experiences from the Princess Máxima Center and UMC Utrecht highlight that success depends on strong stakeholder engagement, clear challenge design, and structured evaluation. While institutional differences may impact outcomes, both challenges led to improved data management practices, awareness, and contributed to new sustainable initiatives.

Looking ahead, embedding data-cleaning challenges into regular institutional workflows can further enhance their impact. Expanding participation through national and international collaborations, integrating data stewardship training, and aligning with broader green ICT policies will be key to scaling up efforts. By fostering a culture of responsible data management, research institutions and hospitals can not only mitigate their digital carbon footprint but also set a precedent for sustainable data practices across disciplines.

## Supporting information

S1 Text Results of the data cleaning challenge at the Princess Máxima Center in Utrecht. The challenge took place in 2023 over a period of one month. The 36 groups of the research department were invited to join the challenge. Data was collected through a digital form and processed using Microsoft Excel. Abbreviations: TB, terabyte; GB, gigabyte.
(DOCX)

S2 Text Results of data cleaning challenge at the University Medical Center in Utrecht. The challenge took place in 2024 over a period of two months. All employees of the institute were invited to join the challenge. Data was collected through a digital form and processed using Microsoft Excel. Abbreviations; TB, terabyte.
(DOCX)

S3 Text Princess Máxima Center Google forms. The text of the digital form that was used to collect results for the data cleaning challenge organized in the Princess Máxima Center.
(DOCX)

S4 Text UMC Utrecht Google forms. The text of the digital form that was used to collect results for the data cleaning challenge organized in the UMC Utrecht.
(DOCX)
