# Supplementary material for: Eleven quick tips for organizing a data cleaning challenge
Source: PLoS Comput Biol. 2025 Dec 16;21(12):e1013791. doi: 10.1371/journal.pcbi.1013791 (PMC12707657; doi:10.1371/journal.pcbi.1013791)
Supplement: S1 Text — The challenge took place in 2023 over a period of one month. The 36 groups of the research department were invited to join the challenge. Data was collected through a digital form and processed using Microsoft Excel. Abbreviations: TB, terabyte; GB, gigabyte. (DOCX) [file pcbi.1013791.s001.docx]

| **Princess Máxima Center** | |
| --- | --- |
| **Data cleaning** | |
| Number of participating research groups | 11 (out of 36) |
| Number of participating individuals | 96 |
| Participating members per group (Av +/- sd) | 55% +/- 26% |
| Total data cleaned | 91.22 TB |
| Surfdrive | 519 GB |
| Personal email | 168 GB |
| Local drive (laptop or PC) | 3.04 TB |
| HPC (shared drive) | 35.21 TB |
| Isilon (shared drive) | 52.28 TB |
| Average storage cleaned per individual | 0.95 TB |
| **Data management** | |
| Data exit policy | 82% of groups |
| Data management plans for running projects | 73% of groups |
| Data record | 73% of groups |
| Folder compressing while cleaning | 45% of groups |
| **Awards** | |
| Largest volume of data cleaned | 56 TB data cleaned by one group |
| Largest relative volume of data cleaned | 46% of data cleaned of total by one group |
| Good data management strategies | The winning group had a data exit policy, data management plans, an active data record, as well as a data steward who actively reminds their colleagues to keep the data record up-to-date |

**S1 Text.** Results of the data cleaning challenge at the Princess Máxima Center in Utrecht. The challenge took place in 2023 over a period of one month. The 36 groups of the research department were invited to join the challenge. Data was collected through a digital form and processed using Microsoft Excel. Abbreviations: TB, terabyte; GB, gigabyte.
