# Supplementary material for: Eleven quick tips for organizing a data cleaning challenge
Source: PLoS Comput Biol. 2025 Dec 16;21(12):e1013791. doi: 10.1371/journal.pcbi.1013791 (PMC12707657; doi:10.1371/journal.pcbi.1013791)
Supplement: S2 Text — The challenge took place in 2024 over a period of two months. All employees of the institute were invited to join the challenge. Data was collected through a digital form and processed using Microsoft Excel. Abbreviations; TB, terabyte. (DOCX) [file pcbi.1013791.s002.docx]

| **University Medical Center Utrecht** | |
| --- | --- |
| **Data cleaning** | |
| Number of participating groups | 15 |
| Number of participating individuals | 50 |
| Total data cleaned | 26.3 TB |
| Personal storage and mailbox | 3.8 TB |
| Other | 22.5 TB |
| Average storage cleaned per individual | 0.5 TB |
| **Awards** | |
| Highest numbers of participating individuals for one group | 11 participants |
| Largest volume of data cleaned | 22.50 TB |
| Highest average data storage reduction per participant from one group | 7.50 TB |
| Largest volume of data cleaned (solely personal storage & mailbox) | 2.49 TB |
| Highest average data storage reduction per participant from one group (solely personal storage & mailbox) | 1.24 TB |

**S2 Text.** Results of data cleaning challenge at the University Medical Center in Utrecht. The challenge took place in 2024 over a period of two months. All employees of the institute were invited to join the challenge. Data was collected through a digital form and processed using Microsoft Excel. Abbreviations; TB, terabyte.
