# Supplementary material for: Eleven quick tips for organizing a data cleaning challenge
Source: PLoS Comput Biol. 2025 Dec 16;21(12):e1013791. doi: 10.1371/journal.pcbi.1013791 (PMC12707657; doi:10.1371/journal.pcbi.1013791)
Supplement: S3 Text — The text of the digital form that was used to collect results for the data cleaning challenge organized in the Princess Máxima Center. (DOCX) [file pcbi.1013791.s003.docx]

**Princess Máxima Center Google forms**

The Green Labs Data Cleaning Challenge aims to green and clean up data & email storage resulting in a reduction of energy usage and CO_2_ emission! Join this challenge by signing up your lab/department/facility. Keep track of your data & email cleaning in this form to gain points and have a chance of being Maxima Digital Cleaner Champions 2023!! It is important that only one responsible person fills the form for the entire group.

1. What is your email-address?
2. What is your team name?

The Green Labs Data Cleaning Challenge 2023 will run during the whole month of October. Teams can decide themselves how to organize the data cleaning, for example by scheduling a day together or you can decide that each team member picks their own cleaning moment(s). We propose each group to have one responsible person to keep track of the cleaning for the whole group in the excel. They add everything together and then fill in this form. Each section of this form has different areas where you can win points, all aimed at reducing data and email storage. Don't worry if you are a small team or a team without "big" data, we will adjust for this when awarding points! For more information you can always email us!

**Email**

Even if you don't work with a lot of data usually, we all use e-mail and after a while of working here you have probably accumulated quite some newsletters, Outlook invites and long e-mail threads. Let's take a look and green up!

If you use a Macbook

Go to Outlook, right-click on your e-mail address in the left hand menu, click 'Properties' -

> 'Storage' and write down the number after 'Total Size (with Subfolders)'.

Please use this format: *a number (if there are decimals, use a point to separate them)* *KB/MB/GB*. For example: 800 KB or 23.4 MB

If you use a Windows computer

Go to Outlook, click 'File' -> 'Tools' -> 'Mailbox Cleanup' -> 'View mailbox size' and write down the number.

Please use this format: *a number (if there are decimals, use a point to separate them)*

*KB/MB/GB*. For example: 800 KB or 23.4 MB

1. What is the size of the email inboxes of all your group members combined *before* cleaning?

After writing down the size, you can filter on big, and old emails and check whether they

can be deleted.

Some other tips regarding email cleaning:

- Unsubscribe from newsletters you don't read anymore.

- Search for sender (for example a newsletter) and delete email.

- Sort emails on size to easily remove your largest emails.

1. What is the size of the email inboxes of all your group members combined *after* cleaning?

**HPC**

If you do not have an HPC account, you can skip to the next section.

For most of us working on the HPC, this will be where most of our data lives and where we can make the biggest difference with data cleaning! Please record the size of your data before cleaning. For the data cleaning itself, remember that you can of course remove things you do not need anymore (or will not need soon and which are easy to regenerate), but you can also compress files or directories. Most software can work directly on gzipped files, so sometimes you can even compress files you regularly use! Another tip: if multiple group members are using the same data, make sure that there are no duplicates!

Log in to the HPC, start up an interactive session (srun --time=0:10:0 --mem=10G --pty

bash), go to your group directory (not your home directory!) and execute the following

command: "du -h -d 1 ."

You will get the size of each of the directories in your directory and at the last line, before

".", you will see the total size of your directory.

1. What is the size of your groups folder/directory on the HPC *before* cleaning?
2. What is the size of your groups folder/directory on the HPC *after* cleaning?

**Princess Máxima internal storage**

Log in to the Maxima server, go to your group directory and execute the following

command: "du -h -d 1 ."

You will get the size of each of the directories in your directory and at the last line, before

".", you will see the total size of your directory.

Also when cleaning the Isilon storage remember that it is possible to compress files or

folders.

1. What is the size of your groups folder/directory on the Princess Máxima internal storage server *before* cleaning?
2. What is the size of your groups folder/directory on the Princess Máxima internal storage server *after* cleaning?

**Surfdrive**

Please record the sum of the size of your data on Surfdrive before cleaning. The size of folders can be found at the right side in and below the column "Size". Also when cleaning the Surfdrive storage remember that it is possible to compress files or

folders.

1. What is the size of your groups folder/directory on surfdrive *before* cleaning?
2. What is the size of your groups folder/directory on surfdrive *after* cleaning?

**Personal local folders**

Have a look at your local personal folders, for example your Downloads folder, and clean when possible. Also here, compressing files and directories is possible!

On MacBook

Click on the folder in your finder then click in the top right corner on the circle with the three dots > Get Info to see the size.

On Windows

Click on the folder in your finder and then click on Properties to see the size.

1. What is the size of your personal local folders *before* cleaning
2. What is the size of your personal local folders *after* cleaning

**Compressing**

1. Did you compress any folders or files during the cleaning?

- Yes
- No
- Other

**Other storage systems**

1. Did you also clean up MS Teams, SharePoint and/or your OneDrive?

- Yes, all three
- Yes, two of them
- Yes, one of them
- No
- Other

**Data management**

Having data management agreements in your lab/department/facility can help organising and reducing data storage! Therefore, you can also win points in this challenge by having some data management policies.

1. Does your group have a data exit policy for when a colleague leaves, that explains what should happen to their data?

- Yes, and we have a system / responsible person in place to manage it
- Yes, but we don’t have a system / responsible person in place to manage it
- No, but we do have unwritten rules about what should happen to data of former
- colleagues
- No, we don't

1. Do you have data management plans (for example on DMP online) in your group?

- Yes, for all projects
- Yes, for most projects
- Yes, for some projects because we recently started doing this
- No, but we will start doing that now
- No, we don't

1. Does your group have a data record, where you keep track of which data has been collected, stored, shared and archived and by whom in the group?

- Yes, it has information for all projects
- Yes, it has information for most projects
- Yes, it has information for some projects because we recently started doing this
- No, but we will start making it now
- No, we don't

**General questions**

1. How many people are in your group?
2. How many people in your group participated in the data cleaning challenge?
3. Have you scheduled a date for your next group data cleaning moment?

- Yes
- No
- No, but we would be happy if regular data cleaning moments are centrally organized by the research department

1. What is something you did that worked really well during the data cleaning, that others can use as well?
2. If there's anything else you would like to mention please do so here (:

Well done! Thank you for participating in the Green Labs Data Cleaning Challenge 2023!

At the beginning of November submission will be reviewed and the Maxima Digital Cleaner Champions 2023 will be announced! Follow the Máxima Green Labs group on intranet to keep up to date on sustainable actions we are taking within our research department and come along to the monthly meetings if you want to work towards a more sustainable research future with us!

For more sustainable actions in the the lab ask us about LEAF for your research group and earn acknowledgement through sustainable actions across your research practices.

**S3 Text.** Princess Máxima Center Google forms. The text of the digital form that was used to collect results for the data cleaning challenge organized in the Princess Máxima Center.
