# Supplementary material for: Eleven quick tips for organizing a data cleaning challenge
Source: PLoS Comput Biol. 2025 Dec 16;21(12):e1013791. doi: 10.1371/journal.pcbi.1013791 (PMC12707657; doi:10.1371/journal.pcbi.1013791)
Supplement: S4 Text — The text of the digital form that was used to collect results for the data cleaning challenge organized in the UMC Utrecht. (DOCX) [file pcbi.1013791.s004.docx]

**UMCU Google forms**

Score sheet Data Cleaning Challenge

Use this form to enter your scores for the Data Cleaning Challenge, which runs from July 1st until August 31st 2024. Good luck!

1. How much data did you delete from your H-drive? (in MBs)
2. How much data did you delete from your mailbox? (in MBs)
3. Got the hang of it and deleted data on other drives as well? Let us know!
4. What is your name?
5. What is your e-mail address?
6. In which team or department do you work? (PLEASE NOTE: everyone should enter the same name to compete for the team prize!)
7. Do you want to become a member of the Green Network at UMC Utrecht?

- Yes please!
- I’m already a member
- No thank you

**S4 Text.** UMC Utrecht Google forms. The text of the digital form that was used to collect results for the data cleaning challenge organized in the UMC Utrecht.
